# Alterations of DNA methylation profile in peripheral blood of children with simple obesity

**DOI:** 10.1007/s13755-024-00275-w

**Published:** 2024-03-18

**Authors:** Yi Ren, Peng Huang, Xiaoyan Huang, Lu Zhang, Lingjuan Liu, Wei Xiang, Liqun Liu, Xiaojie He

**Affiliations:** 1 Department of Pediatrics, The Second Xiangya Hospital of Central South University, Changsha, 410011 China; 2 Children’s Brain Development and Brain Injury Research Office, The Second Xiangya Hospital of Central South University, Changsha, 410011 China; 3 Department of Pediatrics, Haikou Maternal and Child Health Hospital, Haikou, 570100 China; 4 Department of Genetics, Metabolism, and Endocrinology, Hainan Women and Children’s Medical Center, Haikou, 570100 China; 5 Hainan Women and Children’s Medical Center, Haikou, 570100 China; 6 Children’s Hospital of Fudan University at Hainan, Haikou, 570100 China; 7 Children’s Hospital of Hainan Medical University, Haikou, 570100 China; 8 Laboratory of Pediatric Nephrology, Department of Pediatrics, The Second Xiangya Hospital of Central South University, Changsha, 410011 China

**Keywords:** Simple obesity, Children, DNA methylation, Epigenetics

## Abstract

**Purpose:**

To investigate the association between DNA methylation and childhood simple obesity.

**Methods:**

Genome-wide analysis of DNA methylation was conducted on peripheral blood samples from 41 children with simple obesity and 31 normal controls to identify differentially methylated sites (DMS). Subsequently, gene functional analysis of differentially methylated genes (DMGs) was carried out. After screening the characteristic DMGs based on specific conditions, the methylated levels of these DMS were evaluated and verified by pyrosequencing. Receiver operating characteristic (ROC) curve analysis assessed the predictive efficacy of corresponding DMGs. Finally, Pearson correlation analysis revealed the correlation between specific DMS and clinical data.

**Results:**

The overall DNA methylation level in the obesity group was significantly lower than in normal. A total of 241 DMS were identified. Functional pathway analysis revealed that DMGs were primarily involved in lipid metabolism, carbohydrate metabolism, amino acid metabolism, human diseases, among other pathways. The characteristic DMS within the genes Transcription factor A mitochondrial (*TFAM*) and Piezo type mechanosensitive ion channel component 1(*PIEZO1*) were recognized as CpG-cg05831083 and CpG-cg14926485, respectively. Furthermore, the methylation level of CpG-cg05831083 significantly correlated with body mass index (BMI) and vitamin D.

**Conclusions:**

Abnormal DNA methylation is closely related to childhood simple obesity. The altered methylation of CpG-cg05831083 and CpG-cg14926485 could potentially serve as biomarkers for childhood simple obesity.

**Supplementary Information:**

The online version contains supplementary material available at 10.1007/s13755-024-00275-w.

## Introduction

Obesity is a chronic and complex disease caused by abnormal or excessive fat accumulation which has become a significant global public health concern. The prevalence of childhood obesity is increasing every year, and about 206 million children and adolescents aged 5–19 worldwide are projected to have obesity by 2025 [1]. Childhood obesity could lead to various health complications, including vascular endothelial damage, vascular sclerosis, left ventricular diastolic dysfunction, insulin resistance, precocious puberty, kidney injury, asthma, etc. Additionally, childhood obesity could negatively impact cognitive functions such as memory and concentration [2–7]. Overall, obesity has a profound impact on the physical and mental health of children. Moreover, childhood obesity will increase the risk of obesity-related complications in adulthood, such as hyperlipidemia, type 2 diabetes mellitus, hypertension, cardiovascular and cerebrovascular diseases, various cancers, and other diseases [8–10]. These conditions not only diminish the quality of life but also shorten life expectancy. Therefore, early detection and intervention for childhood obesity are crucial to minimize potential complications.

Obesity is a multifactorial disease influenced by both genetic and environmental factors [11]. Epigenetics, as an essential interface of the gene-environment interaction, is closely related to obesity [12]. DNA methylation is the most common epigenetic modification, and abnormal DNA methylation contributes to the initiation and development of multiple diseases, such as metabolic disorders, cardiovascular diseases, and cancer [13]. Previous studies have shown that the DNA methylation profile of children with obesity exhibited hypomethylation compared to normal children [14, 15]. Furthermore, it has been observed that for every 1% increase in mean DNA methylation, the body mass index (BMI) increased by 0.33 kg/m^2^, and the weight increased by 1.16 kg [15]. Compared to the offspring of mothers with a normal weight, the umbilical cord blood from the offspring of mothers with obesity exhibited numerous differential DNA methylated cytosine-phosphate-guanine (CpG) sites [16]. These findings highlight a connection between obesity and DNA methylation and focus primarily on adult obesity. However, the DNA methylation profiles related to childhood obesity remain incompletely understood.

This study aimed to investigate the DNA methylation profile and its alterations in peripheral blood samples from children with simple obesity and normal-weight children. It identified differentially methylated sites (DMS) specifically associated with childhood simple obesity. The results of this study not only contribute to a better understanding of the relationship between DNA methylation and obesity but also could help to recognize potential diagnostic markers and therapeutic intervention targets in childhood obesity.

## Material and methods

### Subjects

The subjects in this study were selected from children (6–14 years) who underwent routine physical examinations at HK Hospital of the Maternal and Child Health from January 2020 to January 2021. The Body Mass Index (BMI) (kg/m^2^) was computed based on their measured body weight (kg) and height (cm).

Considering variations in ethnicity, age, and gender, this study utilized specific BMI thresholds for Chinese children. Children with a BMI ≥ 97th percentile for their age and sex were classified as obese, while those with a BMI between the 15th and 85th percentile were placed in the normal group [17]. Subjects with secondary obesity resulting from metabolic diseases, endocrine conditions, hereditary disorders, other diseases, and glucocorticoid treatment were excluded. Clinical data were collected from the selected subjects.

### Genome-wide DNA methylation analysis

About 3ml venous blood samples were collected from subjects after an overnight fasting of at least 8 h and stored at – 70 °C. Genomic DNA was extracted from peripheral blood lymphocyte samples utilizing a DNA purification kit (QIAGEN, USA) following the manufacturer’s instructions. The Illumina Infinium MethylationEPIC BeadChip (Illumina 850k, San Diego, CA) was applied for analyzing the whole-genome DNA methylation. The minfi *R* package was used to process the original data and calculate the raw beta (*β*) value (*β* = the signal intensity of methylated probes divided by the overall signal intensity). Probes with a detection *p-*value < 0.01 were retained.

To minimize differences within groups and improve data quality, color correction, background adjustment, and quantile normalization were performed. Subsequently, the normalized *β* value was extracted, and the delta *β* (indicating methylation difference) corresponding to each methylated site was calculated by subtracting the *β* value of the methylated site in the obesity group minus in the normal group. The student *t*-test was then applied to the data. Only sites with |delta *β*|> 0.1 and *p*-value < 0.05 were regarded as differentially methylated sites (DMS). A delta *β* < 0 indicates hypomethylation, while delta *β* > 0 means hypermethylation.

### Distributions analysis of DMS regarding gene and CpG island regions

Based on the UCSC annotation, DMS were categorized into specific regions, including the promoter region (transcription start site (TSS) 200, TSS1500, 5′UTR, and 1st Exon), Body, and 3′UTR. Additionally, the DMS was also annotated for CpG island (CGI), CpG shore (within 2kb of CGI), and CpG shelf (within 2 kb-4 kb of CGI).

### Functional pathway analysis of differentially methylated genes (DMG)

Using the ‘clusterprofile R package’ to carry out Gene ontology (GO) enrichment and Kyoto Encyclopedia of Genes and Genomes (KEGG) pathway analysis on DMGs corresponding to the identified DMS.

### Screening of characteristic DMS and DMGs

To identify characteristic DMS, we established the following criteria: (i) the mean *β* value between the obesity and normal group was ≥ 0.6; (ii) the DMS were in or around CGI region; (iii) the DMS were not located on sex chromosomes and were within intergenic regions. Eventually, a total of 18 DMS were recognized. However, due to unsuccessful amplification reactions, only 2 DMS were successfully designed for primer amplification, specifically targeting gene *TFAM* (CpG-cg05831083), and *PIEZO1* (CpG-cg14926485), respectively.

### Pyrosequencing validation of characteristic DMS

The EZ DNA Methylation-Gold kit was applied to transform and purify genomic DNA. Primers of each DMS were designed through PyroMark Assay Design 2.0.2 software (QIAGEN, Inc). Supplementary Table 1 displayed the sequences of primers. The methylation levels of each DMS were calculated using the PyroMark Q48 software (Version 10.6).

### ROC curve analysis of characteristic DMS

To evaluate the predictive value of the characteristic DMS for childhood obesity, the area under the receiver operating characteristic (ROC) curve (AUC) was figured.

### Clinical relevance analysis of characteristic DMS

The correlation between the methylation levels of the characteristic DMS and clinical variables was assessed using the Pearson correlation coefficient (*r*).

### Statistical analysis

Statistical analysis was performed using SPSS version 25.0 (IBM). Descriptive analysis was used for general data. The Mann–Whitney test was employed for continuous variables comparison, while categorical variables were summarized as frequencies and percentages. The χ^2^ test was used for categoric variables. A *p*-value < 0.05 was considered statistically significant.

## Results

### Demographics and clinical features of two groups

A total of 72 subjects were included in the study, with 41 in the obesity group and 31 in the normal. Table 1 provides a summary and comparison of the demographic and specific clinical data between the two groups. The results visualized that the body weight, height, BMI, triglycerides (TG), and uric acid (UA) in the obesity group were significantly higher compared to the normal group, while vitamin D was significantly lower. However, there were no substantial differences between the two groups in terms of gender, age, total cholesterol (TC), low-density lipoprotein (LDL), Ca^2+^, fasting glucose (Glu), thyroid stimulating hormone (TSH), and parathyroid hormone (PTH).

**Table 1:** Demographics and clinical features of two groups

Variables	Obesity group	Normal group	Lower limit of 95% *CI*	Upper limit of 95% CI	*P*-value
Gender (male/female)	20/21	14/17	–	–	0.7607
Age (years)	8.54 ± 1.52	8.14 ± 1.58	− 0.33	1.14	0.2742
Body Weight (kg)	42.93 ± 9.68	27.38 ± 5.62	11.66	19.44	< 0.0001
Height (cm)	137.06 ± 10.90	130.35 ± 9.49	1.81	11.61	0.0079
BMI (kg/m^2^)	22.53 ± 2.44	15.91 ± 1.33	5.66	7.59	< 0.0001
TC (mmol/L)	4.63 ± 0.75	4.64 ± 0.89	− 0.40	0.37	0.9526
TG (mmol/L)	1.28 ± 0.68	0.85 ± 0.29	0.16	0.69	0.0018
LDL (mmol/L)	2.48 ± 0.66	2.22 ± 0.63	− 0.05	0.57	0.0958
Ca^2+^(mmol/L)	2.58 ± 0.20	2.56 ± 0.14	− 0.07	0.11	0.6453
Vit D (nmol/L)	84.56 ± 10.06	90.20 ± 11.57	− 10.73	− 0.54	0.0308
PTH (pmol/L)	1.97 ± 1.70	1.38 ± 0.67	− 0.05	1.24	0.0714
TSH (uIU/ml)	3.21 ± 1.59	3.20 ± 1.29	− 0.69	0.70	0.9812
UA (umol/L)	318.40 ± 66.77	253.90 ± 68.96	32.38	96.67	0.0002
Glu (mmol/L)	4.85 ± 0.62	4.83 ± 0.56	− 0.26	0.30	0.9028

*CI* confidence intervals, *BMI* body mass index, *TG* triglycerides, *UA* uric acid, *Vit D* Vitamin D, *TC* total cholesterol, *LDL* low-density lipoprotein, *Glu* fasting glucose, *TSH* thyroid stimulating hormone, *PTH* parathyroid hormone

### Quality control results of samples and probes

**Fig. 1:**
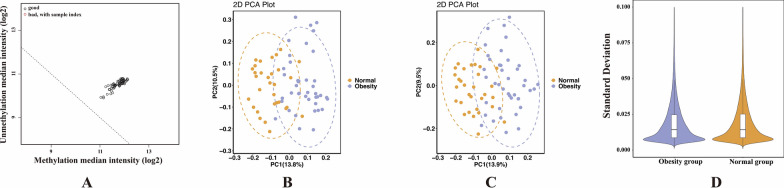
Quality control results of all samples and probes. **A** The horizontal axis represents the log_2_ of methylation median intensity, and the vertical axis represents the log_2_ of unmethylation median intensity. Each circle represents one sample, and the circles at the upper right of the dotted line meet the quality criterion. **B** and **C** represent PCA analysis results with age and gender as covariates, respectively. And the distance between circles reflects the degree of difference in methylation levels among the samples. **D** The violin plot displays the density and range of the standard deviation of methylation level in each group

After normalizing the raw data, the quality of all enrolled samples was assessed. Figure 1A shows that all samples were satisfied with the quality control standards. The PCA analysis indicated incomplete separation between the two sample groups. However, upon introducing age and gender as covariates in PCA analyses, respectively, it was observed that the two groups could be better distinguished (Fig. 1B and C). This observation suggests that there are some DMS between the obesity and normal groups and, age and gender might be important factors influencing DNA methylation levels in childhood obesity. Figure 1D indicates a good internal consistency within the two groups.

### DNA methylation in children with obesity

**Fig. 2:**
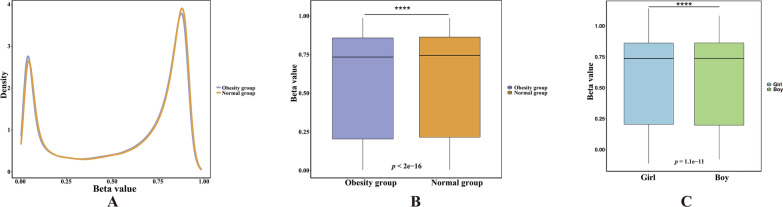
Methylation level distribution. **A** displays the methylation density distribution plot, where the abscissa represents the *β*-value from 0 to 1, and the ordinate represents the frequency of a certain methylation level. **B** Methylation level distribution box plot. **C** Comparison of methylation levels between boys and girls in the obesity group

A total of 865,721 methylated sites were identified in the two groups. The methylation levels in the obesity group and normal group displayed bimodal distribution (Fig. 2A). In comparison to the normal group, the number of unmethylated sites (0 < *β* ≤ 0.2) was greater in the obesity group, and the number of methylated sites (0.8 ≤ *β* ≤ 1) was lower (Fig. 2A). Importantly, the median *β*-value of the obesity group was lower than that of the normal group (*p* < 2e-16) (Fig. 2B), indicating a lower reduced genomic DNA methylation level in the obesity group compared to the normal group. Within the obesity group, the methylation level was lower in boys than in girls (*p* < 0.05) (Fig. 2C).

**Fig. 3:**
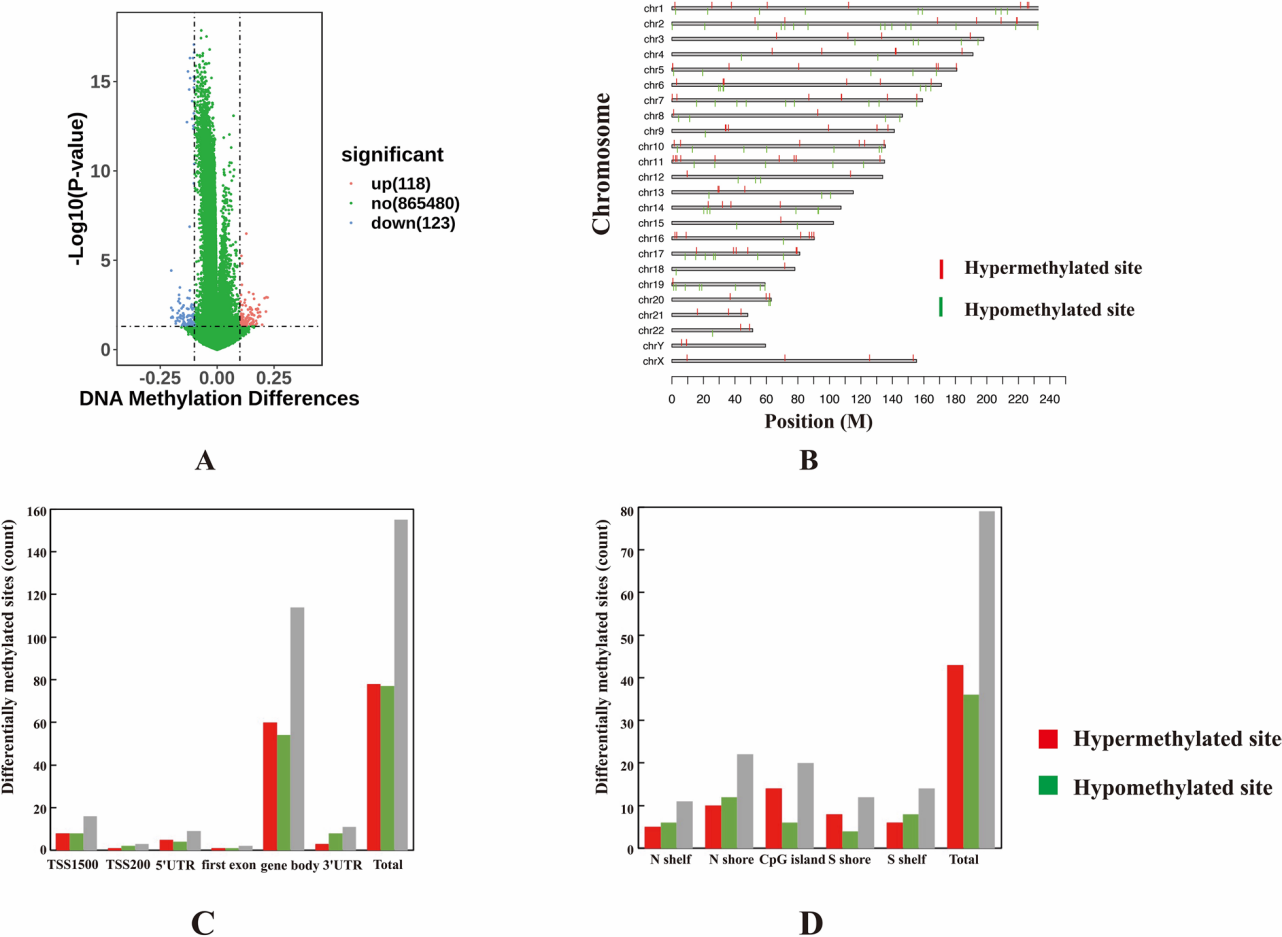
The characteristics of differentially methylated sites in obesity. **A** illustrates the volcano plot of the differentially methylated sites (DMS) based on the DNA methylation differences (delta *β*) and the significance of the difference (*p*-value). Red circles represent differentially hypermethylated sites and blue circles represent differentially hypomethylated sites. **B** The distribution plot showing the locations of DMS on the chromosomes. **C** The distribution of DMS across annotations regardless to the genes. **D**The distribution of DMS concerning CpG islands

Among all the detected methylated sites, we identified 241 differential methylated sites (DMS), comprising 118 hypermethylated sites (delta *β* > 0.1, *p*-value < 0.05) and 123 hypomethylated sites (delta *β* < − 0.1, *p*-value < 0.05) (Fig. 3A), which were associated with 132 genes referred to as differential methylated genes (DMGs). Furthermore, the majority of DMS were found on autosome 6 (Fig. 3B).

### Distribution analysis of DMS in correlation with gene features and CpG island regions

To explore the potential impact of DMS on gene expression, we conducted a comprehensive examination of their distribution. Among the 241 identified DMS, 155 were associated with gene features, including 78 hypermethylated sites and 77 hypomethylated sites, with 78 found in genic regions and the remaining in intergenic regions. Figure 3B illustrates the distribution of DMS in relation to their genomic locations**.** Notably, within the annotated region, the majority of DMS were situated in the gene body region (Fig. 3C). Interestingly, despite an equal count of differentially hypermethylated and hypomethylated sites in the promoter region (encompassing TSS1500, TSS200, 5'UTR, and first exon regions), there was a discernible elevation in methylation levels (mean delta *β* of DMS in the region was 0.0075) as opposed to low methylation (Fig. 3C) (Supplementary Table 2). Furthermore, there are a total of 79 DMS situated within CpG island (CGI) regions, consisting of 43 hypermethylated sites and 36 hypomethylated sites. Figure 3D shows the distribution of DMS in CGI regions, highlighting a notably elevated average methylation level within these genomic loci.

### GO enrichment and KEGG pathway analysis

**Fig. 4:**
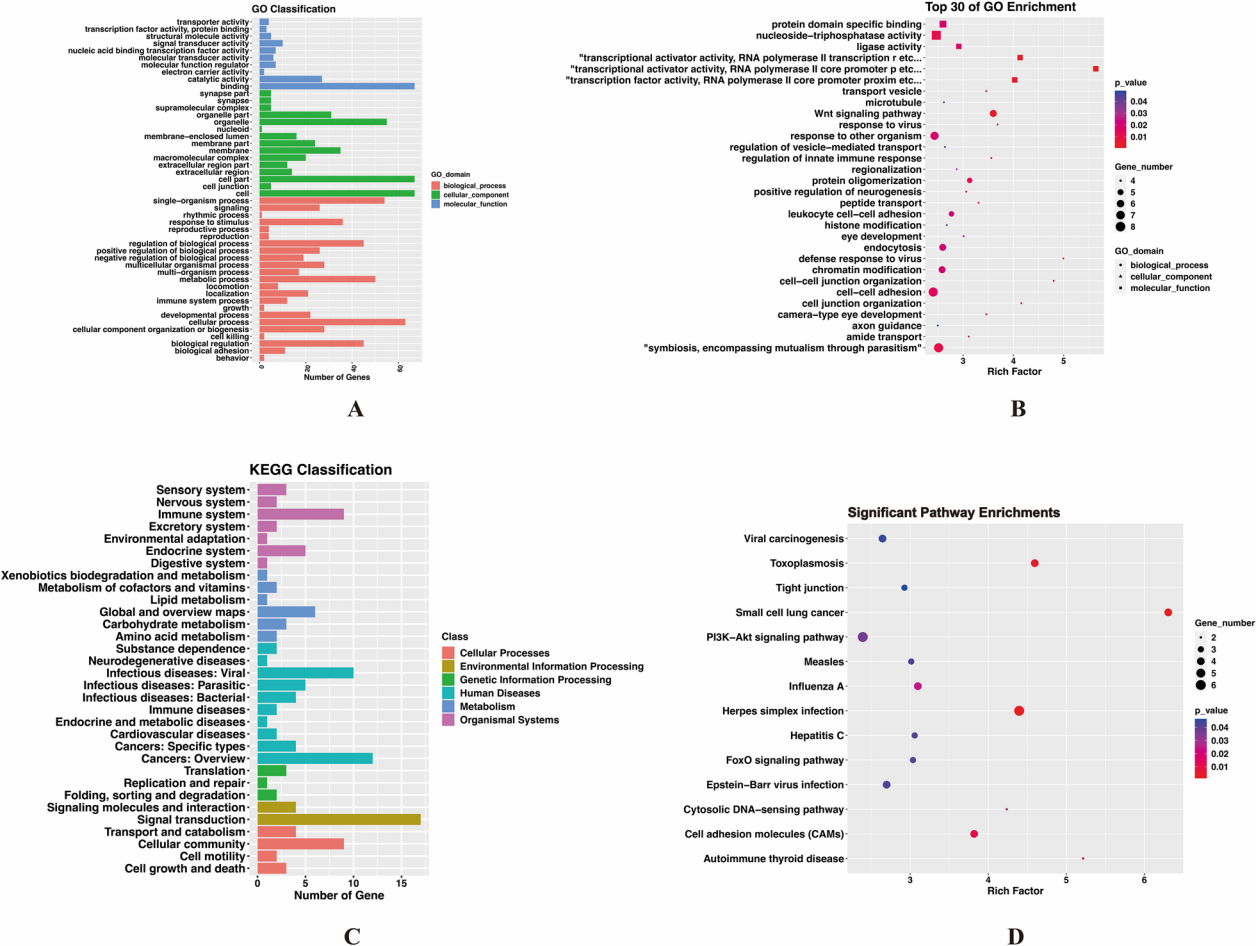
Functional annotations of differentially methylated genes. **A** and **C** show the GO and KEGG classifications, respectively. The abscissa represents the number of genes, while the ordinate represents the categories of GO terms and KEGG pathways, respectively. **B** and **D** display the bubble plots of GO and KEGG enrichment analysis, respectively. The abscissa referring to the rich factors, and the ordinate representing the GO and KEGG items, respectively. The larger the rich factors and the smaller the *p*-value, the more significant the enrichment. A bigger bubble size indicates a higher number of enriched genes

To gain deeper understand the functions of DMGs and the potential mechanistic pathways underlying the onset and progression of obesity, we performed GO enrichment and KEGG pathway analyses. GO enrichment analysis revealed associations with 74 out of the 241 DMGs, with the predominant clusters of GO terms encompassing binding, cell part, cell, cellular process, and organelle. Notably, the top 30 significantly enriched terms included functions such as ATPase activity and T-cell aggregation **(**Fig. 4A and B**)**.

According to the KEGG pathway classification, a total of 51 DMGs were matched to 118 KEGG pathways. The top 3 KEGG pathways were “Environmental Information Processing, Signal transduction”, “Human disease, Cancers: Overview”, and “Organismal System, Endocrine”. Furthermore, KEGG pathway enrichment analysis highlighted 14 pathways with significant enrichment, including Cell adhesion molecules, PI3K-AKT signaling pathway, and FoxO signaling pathway **(**Fig. 4C and D**)**.

### Identification of the characteristic DMGs

**Fig. 5:**
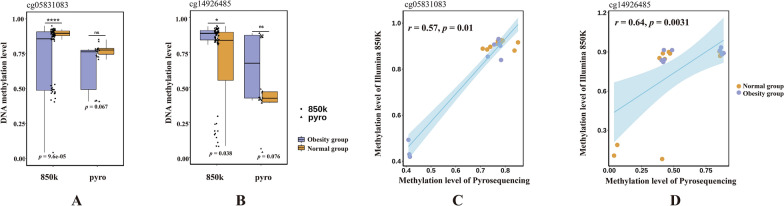
**A** and **B** depict the methylation levels of CpG-cg05831083 and CpG-cg14926485, respectively. The left side of the box plot shows the results from the Illumina 850k chip, while the right presents validation results from pyrosequencing. **C** and **D** illustrate the correlation between pyrosequencing and Illumina 850k chip results. “850k” refers to Illumina 850k chip, and “pyro” refers to pyrosequencing. “*” *p*-value < 0.05; “***” *p*-value < 0.001; “****” *p*-value < 0.0001. “r” represents the Pearson correlation coefficient

According to our filter standards, just 2 DMS were recognized as characteristic: CpG-cg05831083 and CpG-cg14926485, corresponding to the genes *TFAM* and *PIEZO1*, respectively. And the methylation level of CpG-cg05831083 was significantly lower in the obesity group, while CpG-cg14926485 showed significantly higher (Fig. 5A and B).

### Pyrosequencing results of characteristic DMS

To validate the methylation levels of characteristic differentially methylated sites selected from Illumina 850K results, we randomly chose ten obesity cases and ten normal cases from two groups to for pyrosequencing. The results of pyrosequencing demonstrated a high degree of concordance in the methylation level of *TFAM* CpG-cg05831083 and *PIEZO1* CpG-cg14926485 with Illumina 850K results (Fig. 5A and B). Although the observed differences in the methylation levels of CpG-cg05831083 and CpG-cg14926485 between the two groups in the pyrosequencing data were not statistically significant, possibly attributed to the limited sample size, the consistent trends observed reinforce the validity of our findings from another perspective. To further evaluate the reliability of the results, we conducted correlation analyses between Illumina 850K chip data and pyrosequencing data. The correlation analysis revealed a significant positive correlation between the methylation levels of CpG-cg05831083 (*r* = 0.50, *p*-value < 0.05) and CpG-cg14926485 (*r* = 0.62, *p*-value < 0.05) in pyrosequencing results and the corresponding Illumina 850K results (Fig. 5C and D). Considering age and gender as covariates, we were able to clearly distinguish between the normal and obese groups. Consequently, utilizing age and gender as covariates, we separately examined the expression levels of differentially methylated sites. The results indicate that the expression trends of the two differentially methylated sites in both groups align with those observed without covariates (Supplementary Fig. 1). This suggests that these two distinct methylated sites are minimally influenced by age and gender, possibly having little to no impact.

### The AUC of characteristic DMS

**Fig. 6:**
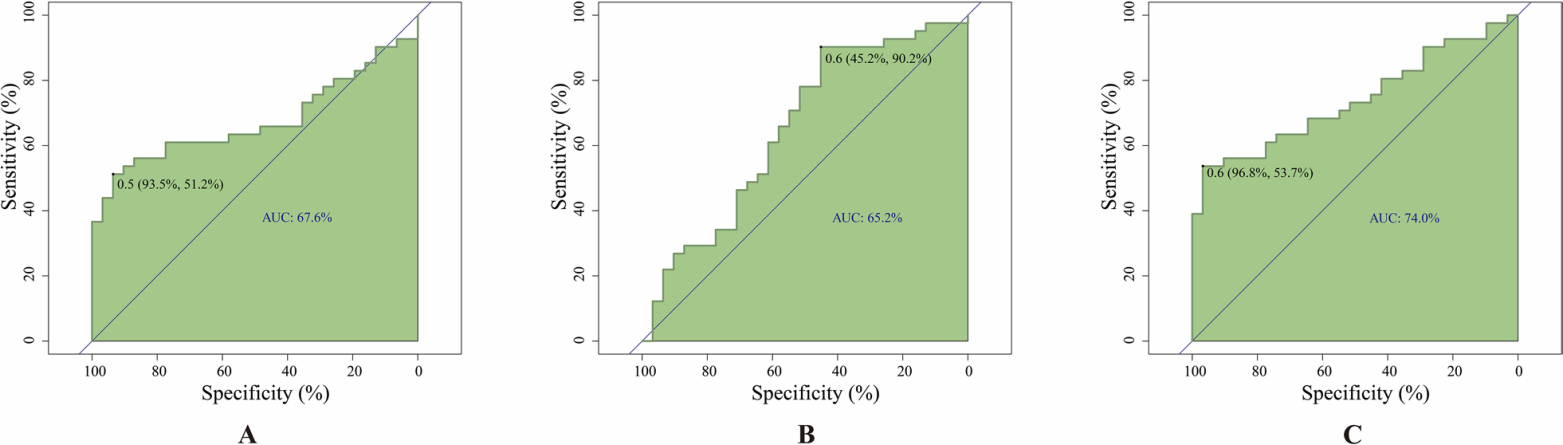
**A** and **B** respectively represent the area under ROC curves (AUC) values for CpG-cg05831083 and CpG-cg14926485. **C** represents the combined AUC when both are considered together

ROC curve analysis based on methylation values reveals the potential value of *TFAM *CpG-cg05831083 and *PIEZO1* CpG-cg14926485 in predicting childhood obesity, with the AUC values of 67.6% and 65.2%, respectively (Fig. 6A and B). Specifically, *TFAM* CpG-cg05831083 shows high specificity and low sensitivity, indicating its accuracy in excluding non-obese individuals but lower accuracy in identifying obese children. Conversely, *PIEZO1* CpG-cg14926485 exhibits high sensitivity and low specificity, suggesting its accuracy in correctly identifying obesity. When considering both sites together, the AUC increases to 74%, with a significant enhancement in specificity to 96.8%, highlighting their synergistic effect (Fig. 6C).

Overall, these findings suggest that methylation patterns of CpG-cg05831083 and CpG-cg14926485 could serve as potential biomarkers for childhood obesity, especially when considered jointly for improved predictive capability.

### Correlations between characteristic DMS and clinical variables

**Fig. 7:**
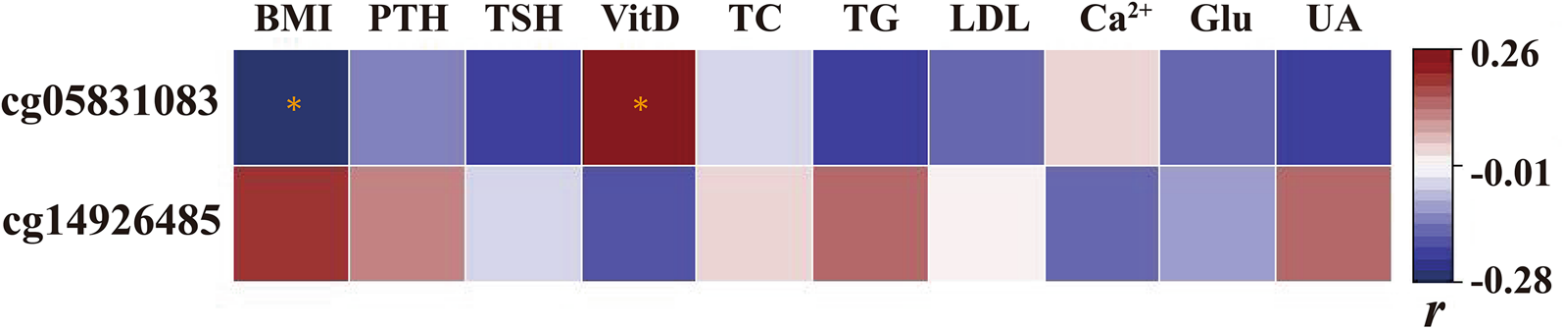
The heatmap represents the correlation between characteristic differentially methylated sites and clinical variables. The blue squares depict negative correlations, while the red squares designate positive ones. The color intensity indicates the strength of the correlations. “r” represents the Pearson coefficient. “*” *p*-value < 0.05

The methylation level of *TFAM* CpG-cg05831083 was remarkably positively related to Vitamin D (*r* = 0.2643, *p*-value < 0.05) and inversely correlated with BMI (*r* = − 0.2822, *p*-value < 0.05). However, the methylation level of *PIEZO1* CpG-cg14926485 showed no significant association with collected clinical indicators (Fig. 7).

## Discussion

Obesity exhibits a strong genetic background, with an estimated heritability of approximately 75% [18]. Up to 60%–80% of the difference in body weight could be explained by heritable factors [18]. The modulation of gene expression crucial to the pathogenesis of childhood obesity, such as fatty acid synthase (Fasn), and fibroblast growth factor-21 gene (FGF21) [19, 20], is intricately linked to DNA methylation in specific genomic regions. Through genome-wide analyses, we found the global DNA methylation level in peripheral blood cells was significantly lower in children with obesity compared to normal controls. Within this context, we identified 241 methylated sites exhibiting differences exceeding 10% between the two groups. Strikingly, only 155 of these DMS were situated in genic regions, while the remaining were distributed across intergenic regions. The human genome consists of intergenic and intragenic regions. During the occurrence and development of diseases, changes in the DNA methylation status in these regions may influence gene expression through actively participating in transcriptional regulation [21–23]. Our research found that childhood obesity is mainly regulated by the DNA methylation of genic regions. A previous study reported that DMS, associated with obesity and epigenetic changes induced by exercise intervention, are enriched in intergenic regions [24]. In our study, we discovered that more than one-third of DMS are in the intergenic region. This implies that many regulatory elements in the intergenic region, such as enhancers and insulators, might also play a role in regulating childhood obesity.

The previous study reported that chronic low-grade inflammation observed in obesity aligns with changes in immune-related pathways [25]. Our study also found the regulation of the innate immune response was highlighted in the GO enrichment analysis, supporting the connection between immune response and obesity. KEGG pathway analysis of DMG reveals that the pathogenesis of obesity is not only related to lipid and carbohydrate metabolism but also to amino acids, cofactors, and vitamin metabolism, which are aspects often overlooked. Additionally, DMG might play a role in complications related to obesity through signaling pathways like PI3K-AKT and FoxO, contributing to issues like insulin resistance, type II diabetes, and cardiovascular disease.

Although genome-wide analysis has recognized numerous genes associated with obesity, the connection between the methylation alterations at specific CpG sites of these genes and obesity, as well as their diagnostic value for obesity remains unclear. This study successfully pinpointed two CpG loci (*TFAM* CpG-cg05831083 and *PIEZO1* CpG-cg14926485) through pyrosequencing in individual samples. The analysis of the methylation levels of CpG-cg05831083 and CpG-cg14926485 in both obese and normal groups will help us understand the role of epigenetic changes in *TFAM* and *PIEZO1* in obesity. Furthermore, ROC curve analysis suggested that CpG-cg05831083 and CpG-cg14926485 could provide better predictions for the occurrence of obesity.

*TFAM* gene is an extremely critical mitochondrial transcription factor [26]. Koh et al. found that specific overexpression of *TFAM* in mice muscles led to increased fatty acid *β*-oxidation, glycolysis, and mitochondrial metabolic rate in muscle tissues. This overexpression was accompanied by the reduction in high-fat diet-induced fat accumulation, insulin resistance, and obesity-induced [27]. Their findings indicate *TFAM* could play a role in regulating the occurrence of obesity. The impact of DNA methylation may depend on the genomic location, and methylation in the gene body tends to be associated with increased gene expression levels [28]. In our study, we identified differentially methylated sites, specifically CpG-cg05831083 located in the gene body region of *TFAM*. This site was significantly hypomethylated in the obesity group. This suggests that the methylation of CpG-cg05831083 may promote *TFAM* expression. If this is the case, *TFAM* expression in obese children is exceedingly likely to be lower than in normal, potentially weakening the function of the *TFAM* gene and ultimately promoting obesity. Our observation exemplifies this, showing a significant negative relationship between the methylation level of CpG-cg05831083 and BMI. Another concern is that the methylation level of CpG-cg05831083 is significantly positively correlated with Vitamin D (Vit D). Vit D, a fat-soluble vitamin, regulates processes such as adipogenesis, immune response, and oxidative stress in mature adipocytes [29]. Increasingly studies have shown an association between Vitamin D deficiency and obesity [29]. Further exploration of the relationship between CpG-cg05831083 methylation and Vitamin D could contribute to a better understanding of the roles of *TFAM* and Vitamin D in the occurrence and progression of obesity.

*PIEZO1* gene encodes the protein which acts as an ion channel, responding to mechanical stimulation by converting it into electrical and chemical signals within cells. This enables cells to respond to changes in membrane tension [30, 31]. Wang et al. [31]. found that *PIEZO1* is highly expressed in mature adipocytes. Knocking out *PIEZO1* in mice fed a high-fat diet, resulted in issues with preadipocyte differentiation into mature adipocytes, larger adipocyte volume, and enlarged inflammation in white adipose tissue. These findings suggested that *PIEZO1*-mediated mechanical signal transduction is involved in adipocyte remodeling and function. The CpG-cg14926485 site was significantly hypermethylated in the obesity group and located in the gene body region. This hypermethylation possibly increases the *PIEZO1* gene expression in childhood with obesity, contributing to its development. However, more studies are needed to uncover the potential mechanisms involved.

There were several limitations in this study. We only explored the DNA methylation profile of peripheral blood. Considering the significant tissue-specific characteristics of DNA methylation, it is necessary to research the DNA methylation profile in other tissues, such as adipose tissue. Additionally, due to a lack of redundant samples, direct links between methylation and gene expression for *TFAM* and *PIEZO1* in obesity and normal groups could not be established. Furthermore, the causal relationship between changes in DNA methylation and obesity remains unclear. Further studies are required to clarify these issues.

## Conclusions

This study revealed the close connection between childhood obesity and DNA Methylation by examining the DNA methylation profile in blood samples from children with simple obesity. The specific CpG sites, CpG-cg05831083 within *TFAM* and CpG-cg14926485 within *PIEZO1* may serve as the potential biomarkers for diagnosing, treating, and preventing childhood simple obesity.

## Supplementary Information

Below is the link to the electronic supplementary material.

Supplementary file1 (JPG 6439 KB)

Supplementary file2 (DOCX 15 KB)

Supplementary file3 (DOCX 20 KB)

## Data Availability

The dataset generated during the current study are available in the NCBI repository (https://www.ncbi.nlm.nih.gov/geo/query/acc.cgi?acc=GSE221864).

## Abbreviations

AUC: Area under the ROC
BMI: Body mass index
BP: Biological process
CC: Cellular component
CGI: CpG island
CpG: Cytosine-phosphate-guanine
DMG: Differentially methylated genes
DMS: Differentially methylated sites
FGF21: Fibroblast growth factor-21 gene
Fasn: Fatty acid synthase
Glu: Fasting glucose
GO: Gene ontology
KEGG: Kyoto encyclopedia of genes and genomes
LDL: Low-density lipoprotein
MF: Molecular function
PCA: Principal component analysis
PIEZO1: Piezo type mechanosensitive ion channel component 1
PTH: Parathyroid hormone
ROC: Receiver operating characteristic curve
TC: Total cholesterol
TFAM: Transcription factor A mitochondrial
TG: Triglycerides
TSH: Thyroid stimulating hormone
TSS: Transcription start site
UA: Uric acid
Vit D: Vitamin D

## References

[1] Jebeile H, Kelly AS, O’Malley G, Baur LA. Obesity in children and adolescents: epidemiology, causes, assessment, and management. Lancet Diabetes Endocrinol. 2022;10:351–65. 35248172 10.1016/S2213-8587(22)00047-X PMC9831747

[2] lAllemand-Jander D. Clinical diagnosis of metabolic and cardiovascular risks in overweight children: early development of chronic diseases in the obese child. Int J Obes (Lond). 2010;34(2):S32–6. 21151144 10.1038/ijo.2010.237

[3] Chen C, et al. Investigating the relationship between precocious puberty and obesity: a cross-sectional study in Shanghai, China. BMJ Open. 2017;7(4): e014004. 10.1136/bmjopen-2016-014004 PMC5566589 28400459

[4] Li M, et al. Predictors of non-alcoholic fatty liver disease in children. Pediatr Res. 2021;92(1):322–30. 34580427 10.1038/s41390-021-01754-6

[5] Yan Y, et al. Child-to-adult body mass index trajectories and the risk of subclinical renal damage in middle age. Int J Obes (Lond). 2021;45(5):1095–104. 33608649 10.1038/s41366-021-00779-5

[6] Sansone F, Attanasi M, Di Pillo S, Chiarelli F. Asthma and obesity in children. Biomedicines. 2020;8(7):231. 32708186 10.3390/biomedicines8070231 PMC7400413

[7] Yanovski JA. Pediatric obesity. An introduction. Appetite. 2015;93:3–12. 25836737 10.1016/j.appet.2015.03.028 PMC4546881

[8] Lee EY, Yoon KH. Epidemic obesity in children and adolescents: risk factors and prevention. Front Med. 2018;12(6):658–66. 30280308 10.1007/s11684-018-0640-1

[9] Strazzullo P, et al. Excess body weight and incidence of stroke: meta-analysis of prospective studies with 2 million participants. Stroke. 2010;41(5):e418–26. 20299666 10.1161/STROKEAHA.109.576967

[10] Renehan AG, et al. Body-mass index and incidence of cancer: a systematic review and meta-analysis of prospective observational studies. Lancet. 2008;371(9612):569–78. 18280327 10.1016/S0140-6736(08)60269-X

[11] Bourdier L, et al. The psycho-affective roots of obesity: results from a french study in the general population. Nutrients. 2020;12(10):2962. 32998238 10.3390/nu12102962 PMC7650670

[12] Jönsson J, et al. Lifestyle Intervention in pregnant women with obesity impacts cord blood DNA Methylation, which associates with body composition in the offspring. Diabetes. 2021;70(4):854–66. 33431374 10.2337/db20-0487 PMC7980200

[13] Elks CE, et al. Variability in the heritability of body mass index: a systematic review and meta-regression. Front Endocrinol (Lausanne). 2012;3:29. 22645519 10.3389/fendo.2012.00029 PMC3355836

[14] Rzehak P, et al. DNA-Methylation and body composition in preschool children: epigenome-wide-analysis in the european childhood obesity project (CHOP)-study. Sci Rep. 2017;7(1):14349. 29084944 10.1038/s41598-017-13099-4 PMC5662763

[15] Zhao J, Goldberg J, Vaccarino V. Promoter methylation of serotonin transporter gene is associated with obesity measures: a monozygotic twin study. Int J Obes (Lond). 2013;37(1):140–5. 22290534 10.1038/ijo.2012.8 PMC3539149

[16] Sharp GC, et al. Maternal pre-pregnancy BMI and gestational weight gain, offspring DNA methylation and later offspring adiposity: findings from the Avon longitudinal study of parents and children. Int J Epidemiol. 2015;44(4):1288–304. 25855720 10.1093/ije/dyv042 PMC4588865

[17] Li H, Ji CY, Zong XN, Zhang YQ. Body mass index growth curves for Chinese children and adolescents aged 0 to 18 years. Chin J Pediatr. 2009;47(07):493–8. 19951508

[18] Wardle J, Carnell S, Haworth CM, Plomin R. Evidence for a strong genetic influence on childhood adiposity despite the force of the obesogenic environment. Am J Clin Nutr. 2008;87(2):398–404. 18258631 10.1093/ajcn/87.2.398

[19] Kim YC, et al. Intestinal FGF15/19 physiologically repress hepatic lipogenesis in the late fed-state by activating SHP and DNMT3A. Nat Commun. 2020;11(1):5969–80. 33235221 10.1038/s41467-020-19803-9 PMC7686350

[20] Yuan X, et al. Epigenetic modulation of Fgf21 in the perinatal mouse liver ameliorates diet-induced obesity in adulthood. Nat Commun. 2018;9(1):636–48. 29434210 10.1038/s41467-018-03038-w PMC5809372

[21] Bell CG, Walley AJ, Froguel P. The genetics of human obesity. Nat Rev Genet. 2005;6(3):221–34. 15703762 10.1038/nrg1556

[22] Murtha M, Esteller M. Extraordinary cancer epigenomics: thinking outside the classical coding and promoter Box. Trends Cancer. 2016;2(10):572–84. 28741488 10.1016/j.trecan.2016.08.004

[23] Zeng X, et al. Genome-wide characterization of host transcriptional and epigenetic alterations during HIV infection of T lymphocytes. Front Immunol. 2020;11:2131. 33013899 10.3389/fimmu.2020.02131 PMC7511662

[24] Rönn T, et al. A six months exercise intervention influences the genome-wide DNA methylation pattern in human adipose tissue. PLoS Genet. 2013;9(6): e1003572. 23825961 10.1371/journal.pgen.1003572 PMC3694844

[25] Emanuela F, et al. Inflammation as a link between obesity and metabolic syndrome. J Nutr Metab. 2012;2012: 476380. 22523672 10.1155/2012/476380 PMC3317136

[26] Ekstrand MI, et al. Mitochondrial transcription factor A regulates mtDNA copy number in mammals. Hum Mol Genet. 2004;13(9):935–44. 15016765 10.1093/hmg/ddh109

[27] Koh JH, et al. TFAM enhances fat oxidation and attenuates high-fat diet-induced insulin resistance in skeletal muscle. Diabetes. 2019;68(8):1552–64. 31088855 10.2337/db19-0088 PMC6692815

[28] Yang X, et al. Gene body methylation can alter gene expression and is a therapeutic target in cancer. Cancer Cell. 2014;26:577–90. 25263941 10.1016/j.ccr.2014.07.028 PMC4224113

[29] Ruiz-Ojeda FJ, Anguita-Ruiz A, Leis R, Aguilera CM. Genetic factors and molecular mechanisms of vitamin D and obesity relationship. Ann Nutr Metab. 2018;73(2):89–99. 29982250 10.1159/000490669

[30] Zarychanski R, et al. Mutations in the mechanotransduction protein PIEZO1 are associated with hereditary xerocytosis. Blood. 2012;120(9):1908–15. 22529292 10.1182/blood-2012-04-422253 PMC3448561

[31] Wang S, et al. Adipocyte Piezo1 mediates obesogenic adipogenesis through the FGF1/FGFR1 signaling pathway in mice. Nat Commun. 2022;11(1):2303. 10.1038/s41467-020-16026-w PMC7211025 32385276

