# Supplementary material for: Alterations of DNA methylation profile in peripheral blood of children with simple obesity
Source: Health Inf Sci Syst. 2024 Mar 18;12(1):26. doi: 10.1007/s13755-024-00275-w (PMC10948706; doi:10.1007/s13755-024-00275-w)
Supplement: Supplementary file 2 — Supplementary file2 (DOCX 15 KB) [file 13755_2024_275_MOESM2_ESM.docx]

**Supplementary Table 1. The primers sequences of Pyrosequencing. “F”: Forward**

**primer, “R”: Reverse primer, “S”: Sequencing primers.**

| **Methylated sites** | **Names of primers** | **Sequences of primers** | **Amplicon sizes (bp)** |
| --- | --- | --- | --- |
| cg14926485 | cg14926485-F | GGTGTGAGGGTATTTGTATAAGG | 178 |
|  | cg14926485-R | CTACAAAAAACCCTCAAAATACATT |  |
|  | cg14926485-S | GGGTTGTAGTGTATATAGGTTGA |  |
| cg05831083 | cg05831083-F | GGTTAGGAGGTTAATAGGAAGGAAAGAA | 156 |
|  | cg05831083-R | AAACCTAACCCTATACAAAATCC |  |
|  | cg05831083-S | AGGTTTAGGTGATTGGTT |  |
