# Supplementary material for: Alterations of DNA methylation profile in peripheral blood of children with simple obesity
Source: Health Inf Sci Syst. 2024 Mar 18;12(1):26. doi: 10.1007/s13755-024-00275-w (PMC10948706; doi:10.1007/s13755-024-00275-w)
Supplement: Supplementary file 3 — Supplementary file3 (DOCX 20 KB) [file 13755_2024_275_MOESM3_ESM.docx]

**Supplementary Table 2. The relevant information regarding differentially methylated sites within the promoter region. “Chr”: Chromosome; “NM”: mRNA accession number.**

| **Target_ID** | **Delta**  **Beta** | **Mean Beta value** | | **P.value** | **Genome Build** | **Chr** | **UCSC Reference gene name** | **UCSC Reference gene accession** | **UCSC Reference gene group** |
| --- | --- | --- | --- | --- | --- | --- | --- | --- | --- |
|  |  | **Obesity** | **Normal** |  |  |  |  |  |  |
| cg13961165 | -0.1187 | 0.5176 | 0.6362 | 0.0000 | GRCh37 | 2 | C2orf27A | NM_013310 | 1stExon |
| cg20256738 | 0.1073 | 0.5931 | 0.4858 | 0.0060 | GRCh37 | Y | TSPY2 | NM_022573 | 1stExon |
| cg18815363 | 0.1283 | 0.8135 | 0.6852 | 0.0000 | GRCh37 | X | TBL1X | NM_001139466 | 5'UTR |
| cg12965540 | -0.1200 | 0.5490 | 0.6690 | 0.0005 | GRCh37 | 5 | MARCH3 | NM_178450 | 5'UTR |
| cg18554395 | -0.1565 | 0.6525 | 0.8090 | 0.0049 | GRCh37 | 19 | GNG7 | NM_052847 | 5'UTR |
| cg06120313 | 0.1049 | 0.6266 | 0.5218 | 0.0126 | GRCh37 | 11 | OSBPL5 | NM_020896 | 5'UTR |
| cg15760241 | -0.1024 | 0.7756 | 0.8780 | 0.0131 | GRCh37 | 22 | LRP5L | NM_001135772 | 5'UTR |
| cg08014404 | 0.1052 | 0.8212 | 0.7159 | 0.0217 | GRCh37 | 11 | LRP5 | NM_001291902 | 5'UTR |
| cg07506503 | 0.1978 | 0.7150 | 0.5171 | 0.0403 | GRCh37 | 9 | KIF24 | NM_194313 | 5'UTR |
| cg26757229 | -0.1003 | 0.5250 | 0.6253 | 0.0448 | GRCh37 | 11 | BIRC3 | NM_182962; | 5'UTR |
| cg21886864 | 0.1184 | 0.7622 | 0.6437 | 0.0458 | GRCh37 | 11 | ODZ4 | NM_001098816 | 5'UTR |
| cg22682254 | -0.1047 | 0.5287 | 0.6333 | 0.0000 | GRCh37 | 17 | CRYBA1 | NM_005208 | TSS1500 |
| cg24963067 | -0.1024 | 0.4890 | 0.5914 | 0.0000 | GRCh37 | 14 | OR4M1 | NM_001005500 | TSS1500 |
| cg14459203 | 0.2160 | 0.8027 | 0.5867 | 0.0012 | GRCh37 | 11 | CCDC34 | NM_080654 | TSS1500 |
| cg23024553 | 0.2218 | 0.7973 | 0.5754 | 0.0012 | GRCh37 | 11 | CCDC34 | NM_080654 | TSS1500 |
| cg20832302 | -0.1141 | 0.2980 | 0.4121 | 0.0037 | GRCh37 | 2 | DYSF | NM_001130983 | TSS1500 |
| cg03551377 | -0.1085 | 0.6696 | 0.7781 | 0.0057 | GRCh37 | 2 | IMMT | NM_001100170 | TSS1500 |
| cg16119776 | 0.2144 | 0.6065 | 0.3921 | 0.0074 | GRCh37 | 5 | RARS | NM_002887 | TSS1500 |
| cg11828654 | 0.1099 | 0.2662 | 0.1563 | 0.0117 | GRCh37 | X | TMEM187 | NM_003492 | TSS1500 |
| cg19759212 | -0.1008 | 0.6963 | 0.7970 | 0.0164 | GRCh37 | 1 | TSACC | NM_144627; | TSS1500 |
| cg24760581 | -0.1508 | 0.2388 | 0.3896 | 0.0194 | GRCh37 | 6 | HLA-DRB1 | NM_002124 | TSS1500 |
| cg20427171 | -0.1093 | 0.5117 | 0.6210 | 0.0268 | GRCh37 | 8 | TSTA3 | NM_003313 | TSS1500 |
| cg11475788 | 0.1091 | 0.6986 | 0.5895 | 0.0284 | GRCh37 | 11 | CEND1 | NM_016564 | TSS1500 |
| cg26954695 | -0.1818 | 0.4070 | 0.5888 | 0.0344 | GRCh37 | 19 | MZF1 | NM_003422 | TSS1500 |
| cg10946263 | 0.1058 | 0.8346 | 0.7289 | 0.0375 | GRCh37 | 5 | C5orf33 | NM_153013 | TSS1500 |
| cg04513561 | 0.1218 | 0.7867 | 0.6649 | 0.0439 | GRCh37 | 3 | TP63 | NM_001114982 | TSS1500 |
| cg10224537 | 0.1285 | 0.5597 | 0.4312 | 0.0465 | GRCh37 | 2 | B3GALT1 | NM_020981 | TSS1500 |
| cg13312387 | -0.1022 | 0.1139 | 0.2161 | 0.0032 | GRCh37 | 2 | NMUR1 | NM_006056 | TSS200 |
| cg04422742 | -0.2016 | 0.2842 | 0.4858 | 0.0046 | GRCh37 | 6 | HLA-DRB6 | NR_001298 | TSS200 |
| cg10890644 | 0.1090 | 0.1665 | 0.0575 | 0.0190 | GRCh37 | 10 | TUBAL3 | NM_024803 | TSS200 |
